# [Retracted] Formyl peptide receptor 2 expression predicts poor prognosis and promotes invasion and metastasis in epithelial ovarian cancer

**DOI:** 10.3892/or.2025.8922

**Published:** 2025-06-04

**Authors:** Xiaohui Xie, Mengyuan Yang, Yiling Ding, Ling Yu, Jianlin Chen

Oncol Rep 38: 3297-3308, 2017; DOI: 10.3892/or.2017.6034

Following the publication of this paper, it was drawn to the Editor's attention by a concerned reader that the authors had apparently compiled the Transwell assay data shown in Fig. 5C on p. 3304 incorrectly. Subsequently, the Editorial Office performed an independent analysis of the data shown in this paper, and this investigation revealed that certain of the tube formation assay data shown in Fig. 6 had apparently appeared in an article published in the journal *Molecular Medicine Reports* a few years previously, which had been written by different authors at different institutes. Owing to the fact that the contentious data in the above article had already been published prior to its submission to *Oncology Reports*, the Editor has decided that this paper should be retracted from the Journal. The authors were asked for an explanation to account for these concerns, but the Editorial Office did not receive a reply. The Editor apologizes to the readership for any inconvenience caused.

